# The impact of multivisceral liver resection on short- and long-term outcomes of patients with colorectal liver metastasis: A systematic review and meta-analysis

**DOI:** 10.1016/j.clinsp.2022.100099

**Published:** 2022-09-17

**Authors:** Sérgio Silveira Júnior, Francisco Tustumi, Daniel de Paiva Magalhães, Vagner Birk Jeismann, Gilton Marques Fonseca, Jaime Arthur Pirola Kruger, Fabricio Ferreira Coelho, Paulo Herman

**Affiliations:** a Divisão de Cirurgia do Aparelho Digestivo, Departamento de Gastroenterologia, Hospital das Clínicas, Faculdade de Medicina, Universidade de São Paulo (HCFMUSP), São Paulo, SP, Brazil; b Instituto do Câncer do Estado de São Paulo (ICESP), Hospital das Clínicas, Faculdade de Medicina, Universidade de São Paulo (HCFMUSP), São Paulo, SP, Brazil

**Keywords:** Colorectal neoplasms, Liver, Hepatectomy, Margins of excision, Survival analysis, Meta-analysis

## Abstract

The impact of Multivisceral Liver Resection (MLR) on the outcome of patients with Colorectal Liver Metastasis (CRLM) is unclear. The present systematic review aimed to compare patients with CRLM who underwent MLR versus standard hepatectomy regarding short- and long-term outcomes. MLR is a feasible procedure but has a higher risk of major complications. MLR did not negatively affect long-term survival, suggesting that an extended resection is an option for potentially curative treatment for selected patients with CRLM.

## Highlights

• Multivisceral liver resections have acceptable long-term oncologic outcomes.
• Multivisceral liver resections are safe. They have similar rates of blood loss and postoperative complications.
• Multivisceral liver resections are longer and may be associated with a longer length of hospital stay.

## Introduction

The incidence of colorectal cancer has been increasing in recent decades, reaching more than 1,930,000 newly diagnosed cases in 2020.^1^,^2^ Currently, colorectal cancer is the second most frequent cause of cancer-associated mortality worldwide with approximately 935,000 deaths annually.^2^ The liver is the most common site of metastatic spread (up to 80% of patients).^3^ Approximately 50% of patients with colorectal cancer will develop liver metastases during follow-up, and 15% to 25% of these patients will be diagnosed with their primary tumors.^3^,^4^ The incidence of Colorectal Liver Metastasis (CRLM) is approximately 4.3% in 1-year, 8.7% in 2-years, 12.7% in 3-years, and 16.5% in 5-years.^5^ Importantly, the metastatic disease has a significant prognostic impact, accounting for two-thirds of deaths in patients with colorectal cancer.^6^

The cornerstone of CRLM treatment is the combination of systemic chemotherapy and complete resection of liver lesions with clear surgical margins (R0 resection), resulting in a 5-year Overall Survival (OS) of 40%‒60%.^7^, ^8^, ^9^ However, the lesions of only 20%‒25% of patients with CRLM are considered resectable at initial presentation.^10^

Surgical margins are a major issue in the surgical treatment of CLRM. Several studies have shown that microscopic-free surgical margins offer long-term benefits compared to R1 resections.^11^, ^12^, ^13^, ^14^, ^15^, ^16^ Therefore, for patients with locally advanced CRLM involving adjacent organs or structures, hepatectomy combined with resection of the involved adjacent organs/structures is necessary to achieve free surgical margins.^17^

However, the impact of Multivisceral Liver Resection (MLR) on patients with CLRM who underwent surgical treatment is unclear. Some studies have shown a negative impact of multi-visceral resections on perioperative morbidity and significantly worse long-term outcomes,^18^,^19^ while other studies have failed to detect any difference comparing MLR and standard hepatectomy.^17^,^20^ Despite the increased performance, the available evidence that supports MLR in patients with CRLM is from retrospective cohorts^19^,^20^ and comparative studies with underpowered small sample sizes.^17^, ^18^, ^19^, ^20^ To date, no systematic review or meta-analysis has been published on this topic, indicating that quality data supporting the indications, feasibility, and oncological outcomes of MLR are lacking.

The present study aimed to compare the short- and long-term outcomes of patients with CRLM who underwent MLR versus standard hepatectomy with curative intent.

## Methods

The present study was approved by the Institutional Ethics Committee and conducted following the Preferred Reporting Items for Systematic Reviews and Meta-Analyses (PRISMA) guidelines.^21^ This research protocol was registered in the International Prospective Register of Systematic Reviews (http://www.crd.york.ac.uk/PROSPERO) under number CRD42021244265.

### Database search

A systematic review was performed in PubMed, Embase, Cochrane Library Central, Scientific Library Electronic Online/Latin American and Caribbean Health Sciences Literature (SciELO/LILACS), and grey literature by two independent authors. Databases were searched for Randomized Controlled Trials (RCTs) and comparative observational studies that evaluated the perioperative and long-term outcomes of patients who underwent MLR or standard hepatectomy for CRLM with curative intent. The search was limited to human subjects and included prospective and retrospective studies regardless of language or date of publication. Retrieved references were cross-checked manually for additional studies. The last search was performed on June 09, 2022.

The search strategy in PubMed was based on the following Medical Subject Headings (MeSH) and keywords: ((((multivisceral) OR (extended) OR (diaphragm) OR (stomach) OR (gastric) OR (gastrectomy) OR (inferior vena cava) OR (kidney) OR (nephrectomy)) AND (((hepatectomy) OR (hepatectomies) OR (liver resection)) AND (((colorectal) OR (rectal) OR (colonic)) AND ((neoplasm) OR (cancer) OR (tumour) OR (carcinoma) OR (adenocarcinoma)))))). For EMBASE, Cochrane Library Central, and SciELO/LILACS, the search was performed with the same keywords in various combinations.

### Study selection

The study selection was performed by two independent reviewers. Any disagreement on the inclusion or exclusion of a given study was resolved by a consensus meeting. Initially, titles and abstracts were screened, and irrelevant (or duplicate) studies were excluded; the full text of potentially eligible articles was then analyzed. The following inclusion criteria were used: (1) RCTs and observational studies (prospective or retrospective) that compared perioperative and/or long-term outcomes of patients with CRLM who underwent MLR or standard hepatectomy; and (2) The definition of MLR was any hepatectomy with en bloc resection of at least one adjacent organ or structure, including extrahepatic vascular resections (e.g., Inferior Vena Cava [IVC] and/or hepatocaval confluence) not usually performed in a standard hepatectomy. Associated resection of the gallbladder, hepatic pedicle structures (hepatic artery, portal vein, and biliary tree), and simultaneous resection of gastrointestinal tumors and synchronous liver metastasis without direct invasion of the liver by the primary tumor was not considered MLRs.^22^ If the same patients were included in more than one study, the most recent or the one of higher quality was selected.

The exclusion criteria were as follows: (1) Noncomparative studies, review articles, letters, and case reports; (2) Studies with other definitions of MLRs; (3) Studies with missing values or data for outcome calculation; and (5) Studies unavailable in full text.

### Data extraction

Full text, tables, and figures of selected studies were assessed for data extraction. The following data were collected: (1) Name of the first author and year of publication; (2) Study type; (3) Number of patients per group; (4) Patient characteristics, including age and sex; (5) Type of liver resection and type and number of adjacent organs/structures resected; and (6) Outcomes, including operative time, estimated blood loss, blood transfusion rate, length of hospital stay, frequency of compromised margins, overall morbidity, and 30-day perioperative mortality. Perioperative morbidity was stratified according to the Clavien-Dindo classification.^23^

### Level of evidence and quality assessment

Study quality was assessed using Robins-I,^24^ and certainty assessment was performed using Grading of Recommendations Assessment, Development, and Evaluation (GRADE) recommendations.^25^

### Statistical analysis

The meta-analysis was performed using STATA 16.1 software. Continuous variables are expressed as the mean ± SD and summarized as the Mean Difference (MD) and 95% Confidence Interval (95% CI). Categorical variables are expressed as absolute numbers and summarized as Risk Differences (RDs) and 95% CIs. Hazard Ratios (HRs) and their corresponding lower and upper 95% CI limits were extracted from the individual time-to-event outcomes of the included studies. When the HR and associated standard error or CI were not provided, the HR was calculated using different statistical methods based on the clinical and statistical data reported in the primary studies.^26^,^27^

Study heterogeneity was assessed using chi-square and I^2^ statistics. A random-effects analysis model was applied to adjust for expected interstudy heterogeneity to provide a more conservative CI around the pooled HR.^28^ Because no more than ten studies were included in the meta-analysis, publication bias evaluation was not performed due to the low power of the funnel plot test to distinguish chance from real asymmetry.

Whenever possible, subgroup analysis according to the type of extrahepatic organ/structure resected was performed. The significance level was set at 5% (p < 0.05).

## Results

### Baseline characteristics

**Fig. 1:**
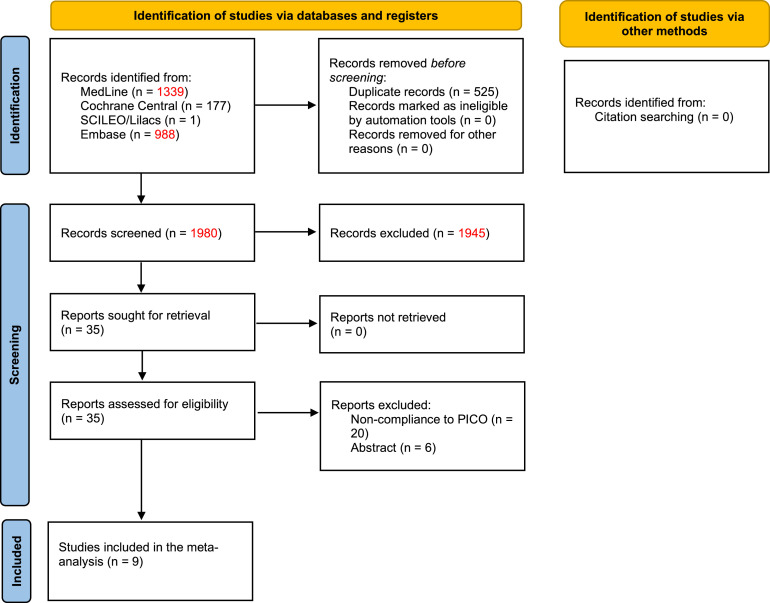
Flowchart of the search strategy and study selection.

Of the 1,980 initially screened articles, 9 comparative studies (comprising 1,786 patients) were included in the systematic review (Fig. 1).^17^, ^18^, ^19^, ^20^^,^^22^^,^^29^, ^30^, ^31^, ^32^ All of the included studies were observational, and no RCTs were found. A previous case-match study published by the present group compared the outcomes of MLR vs. standard hepatectomy; however, the CRLM subgroup was evaluated only for long-term outcomes.^22^ The raw data of this subgroup were retrieved and included in the quantitative analyses of perioperative outcomes. The baseline characteristics of the included studies are shown in Table 1. The assessment of certainty and risk of bias are shown in Supplementary Files 1 and 2, respectively.

**Table 1:** Baseline characteristics of the included studies.

		Standard hepatectomy					Multivisceral liver resection					
Author	Year	Age (years)	Male (%)	Median follow-up	Operative approach	n	Age (years)	Male (%)	Median follow-up	Operative approach	n	Extra-hepatic resection
Hand et al.	2018	69	56	41	21 RH; 6 LH 30 Segmentectomy	57	65	58	41	7 RH; 2 LH; 10 Segmentectomy	19	13 Diaphragm; 4 Inferior vena cava; 1 Kidney/adrenal; 1 Small bowel; 1 Psoas muscle
Shinke et al.	2018	63	67	> 60	NI	158	65	60	> 60	NI	20	10 Diaphragm; 5 Inferior vena cava; 1 Kidney/adrenal; 1 Small bowel; 1 Pericardium; 1 Abdominal wall; 1 Biliary tree
Silveira Jr et al.	2020	59	57	22	27 Major; 41 Minor	68	64	60	21	11 Major; 12 Minor	23	8 Diaphragm; 3 Stomach; 3 Duodenum; 1 Small bowel; 2 Kidney/adrenal; 5 Inferior vena cava; 2 Colon
Li et al.	2012	60	59	22	177 Major; 81 RH; 41 LH; 16 Segmentectomy	408	55	59	22	20 Major; 12 RH; 1 LH; 1 Segmentectomy	34	34 Diaphragm
Lordan et al.	2009	66	66	34	NI	258	67	69	34	NI	27	27 Diaphragm
Kazaryan et al.	2020	69	59	26	25 RH; 12 LH; 368 Non-anatomic; 50 Segmentectomy	455	66	66	31	3 Major; 9 Segmentectomy	12	12 Diaphragm
Lainas et al.	2015	64	62	36	45 RH	45	63	57	36	7 RH	7	7 Diaphragm
Johnson et al.	2006	NI	NI	33	NI	97	60	36	33	4 RH; 5 Trisectionectomy; 2 RH	11	11 Inferior vena cava
Aoki et al.	2004	63	62	26	11 Major; 67 Minor	78	55	66	26	6 Major; 3 Minor	9	3 Inferior vena cava; 6 hepatic venous confluence

NI, Not Informed; RH, Right Hepatectomy; LH, Left Hepatectomy.

The mean age was 64 years with a male predominance (61%), and the mean postoperative follow-up was 31 months. Subgroup analysis was possible for patients who underwent associated diaphragm resection (4 studies, n = 1,246 patients),^18^,^19^,^29^,^31^ associated vascular resection (2 studies, n = 195 patients),^30^,^32^ and other MLRs (3 studies, n = 345 patients).^17^,^20^,^22^

### Short-term outcomes

**Fig. 2:**
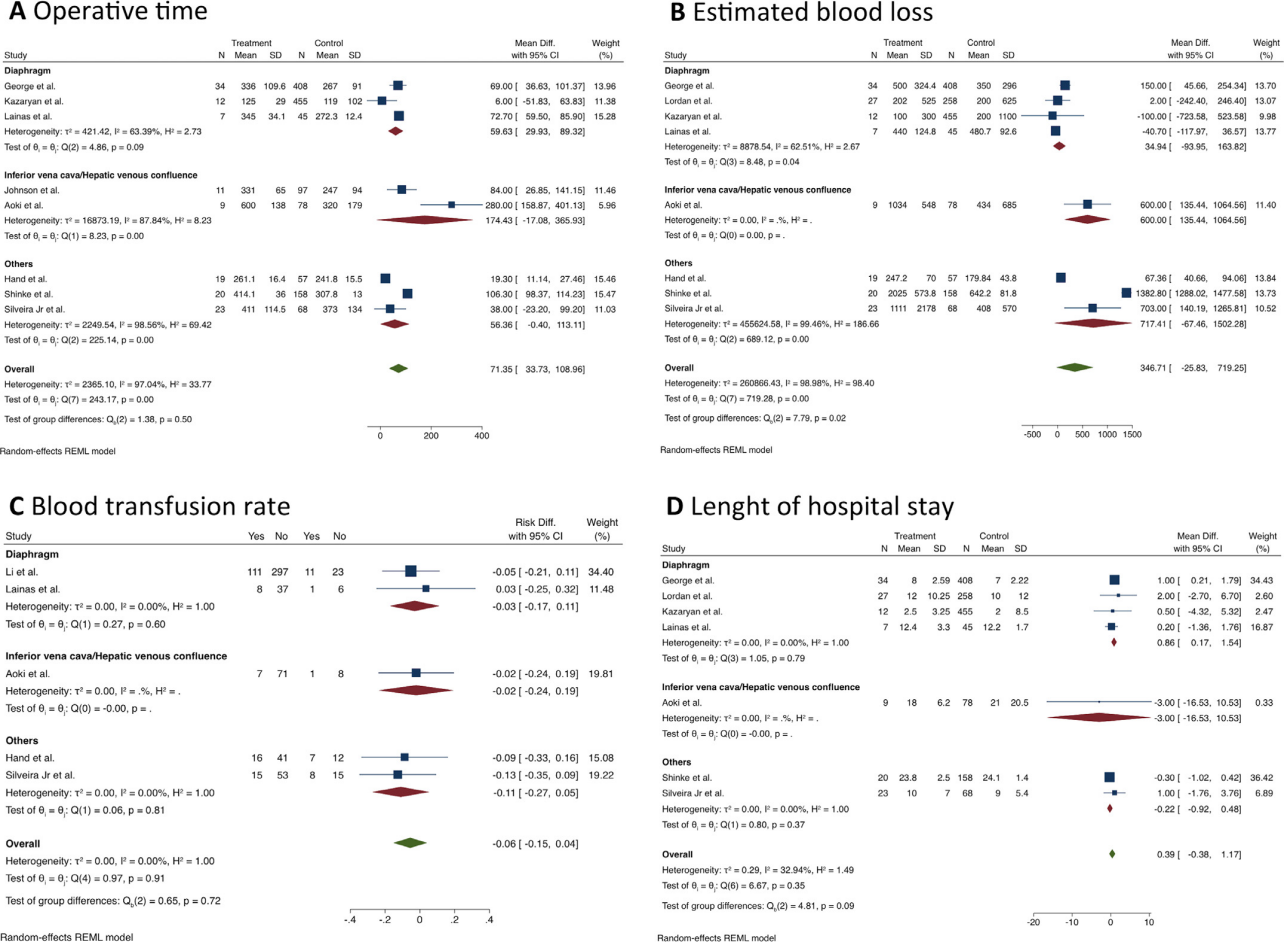
Forest plots depicting (A) operative time, (B) estimated blood loss, (C) blood transfusion rate, and (D) length of hospital stay (multivisceral liver resection vs. standard hepatectomy).

Patients who underwent MLR had longer operative times than standard hepatectomy (MD = 71.4 min; 95% CI 33.7 to 109; I^2^ = 97%; 8 studies; 1,501 patients; the certainty of evidence: low). The same finding was observed in the diaphragm resection subgroup (MD = 59.6 min; 95% CI 30.0‒89.4; I^2^ = 63.4%, Fig. 2A).

Estimated blood loss was assessed in 8 studies (n = 1,678 patients) (Fig. 2B), and the pooled analysis showed no difference between the groups (MD = 346.7 mL; 95% CI -25.8 to 719.3; I^2^ = 99%; certainty of evidence: very low). Similarly, no difference was found in the blood transfusion rate (RD = 0.06; 95% CI -0.04 to 0.15; I^2^ = 0%; certainty of evidence: very low, Fig. 2C).

Seven articles (n = 1,602 patients) reported results concerning the length of hospital stay (Fig. 2D), and no significant difference was found between the groups (MD = 0.39 days; 95% CI -0.38 to 1.17; I^2^ = 33%; certainty of evidence: moderate). However, MLR was associated with a longer hospital stay in the subgroup of patients who underwent diaphragm resection (MD = 0.86 days; 95% CI 0.17 to 1.54; I^2^ = 0%).

MLR was not associated with a higher risk for postoperative complications (RD = -0.01; 95% CI -0.12 to 0.09; I^2^ = 30%; 8 studies; 1,678 patients; certainty of evidence: moderate). However, analysis of the perioperative complications according to the Clavien-Dindo classification indicated that the MLR group had a higher rate of major complications (Grade III‒IV) (RD = 0.07; 95% CI 0.01 to 0.13; I^2^ = 0%; 5 studies, 484 patients; the certainty of evidence: moderate), but no differences were found in the subgroup analysis.

The reported perioperative mortality ranged from 0 to 7.4% in the MLR group and from 0 to 3% in the standard hepatectomy group (9 studies, n = 1,786 patients) with no difference between the groups (RD = 0.00; 95% CI -0.00 to 0.01; I^2^ = 0%; the certainty of evidence: moderate).

**Fig. 3:**
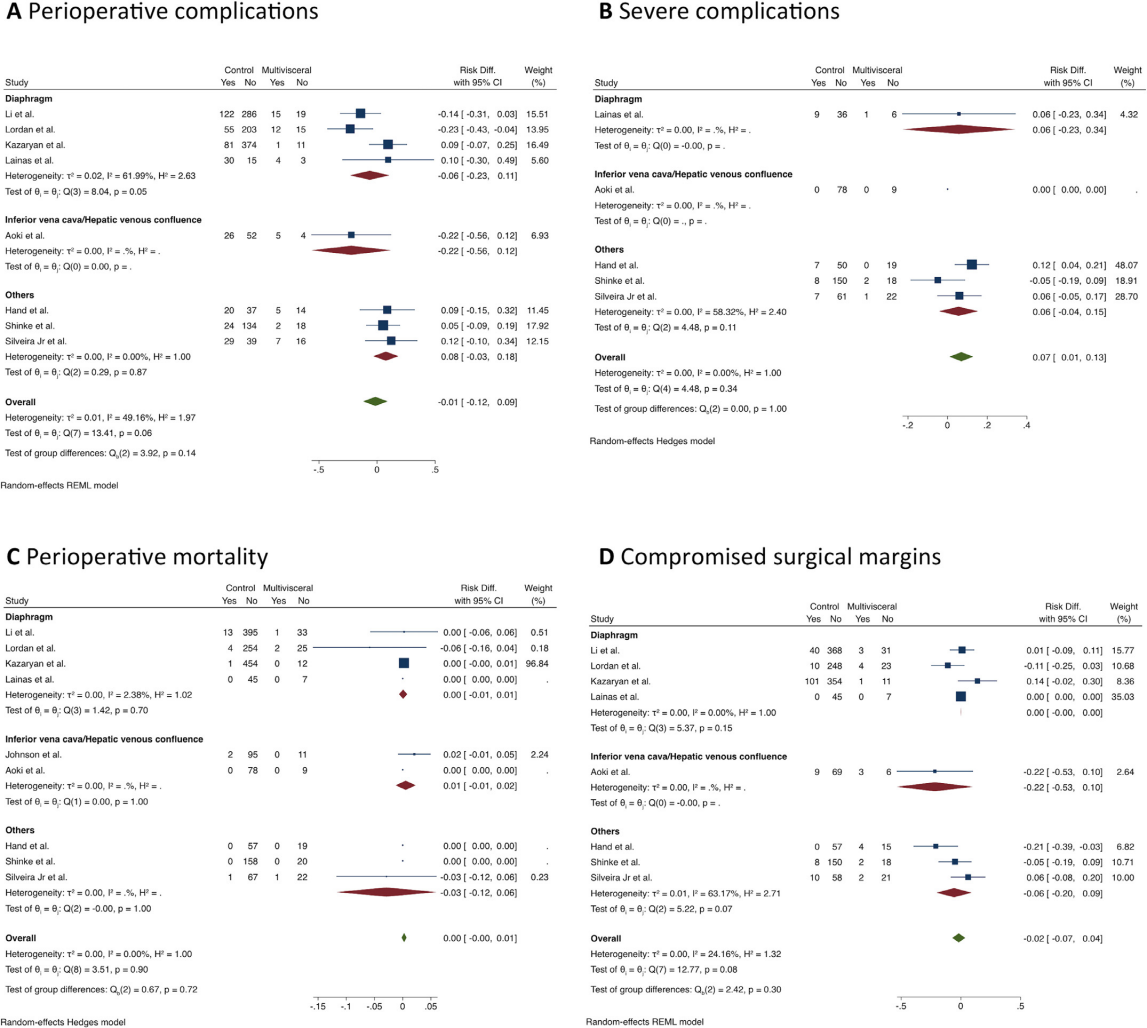
Forest plots depicting (A) perioperative complications, (B) major complications (according to Clavien-Dindo classification), (C) perioperative mortality, and (D) compromised surgical margins (multivisceral liver resection vs. standard hepatectomy).

The frequency of compromised margins was also similar between the groups (RD = 0.02; 95% CI -0.04 to 0.07; I^2^ = 24%; 8 studies, 1,768 patients; the certainty of evidence: moderate), (Fig. 3).

### Long-term outcomes

**Fig. 4:**
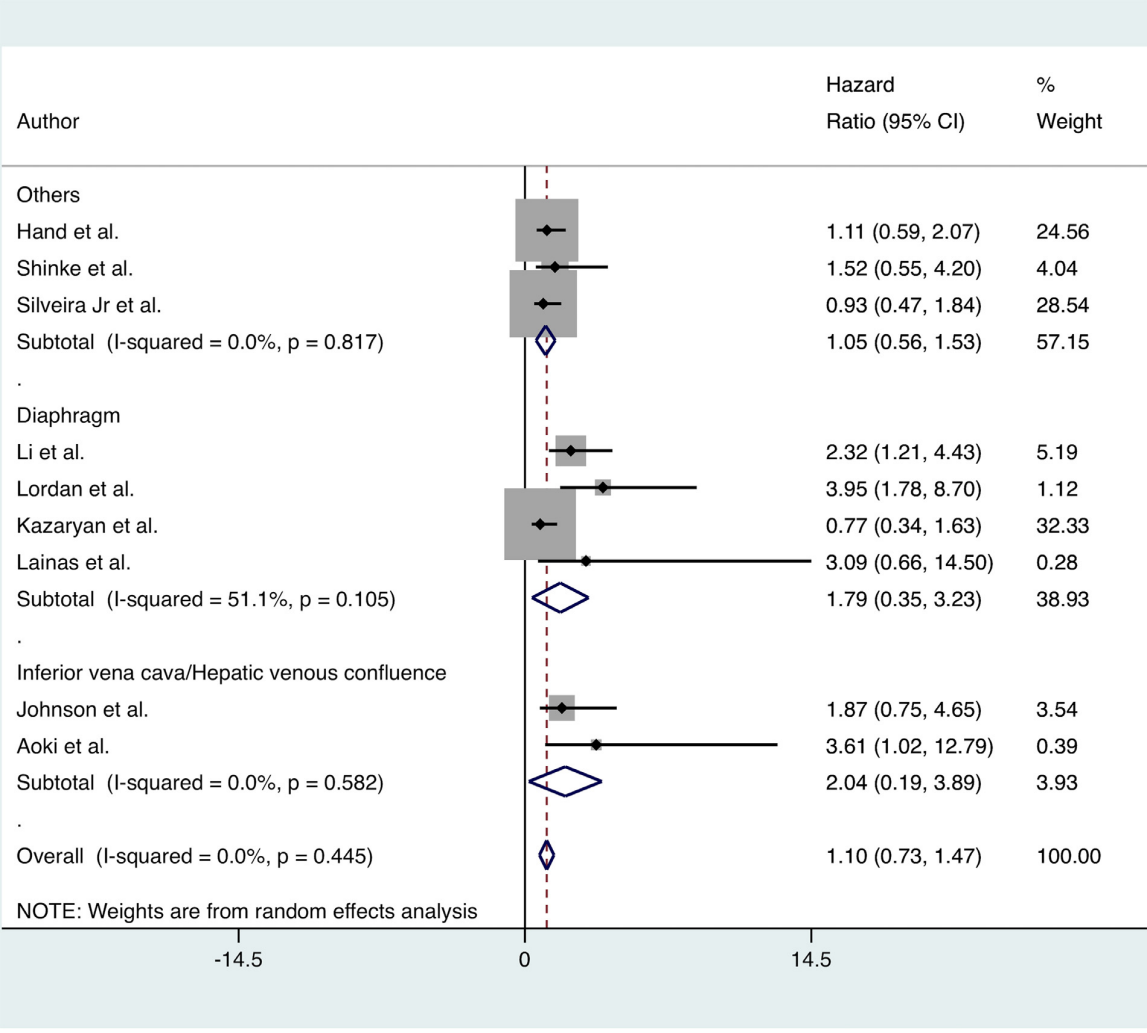
Forest plots depicting overall survival in the multivisceral liver resection vs. standard hepatectomy groups.

No significant difference in OS was found between the MLR and standard hepatectomy groups (HR = 1.10; 95% CI 0.73 to 1.47; I^2^ = 0%; 9 certainties of evidence: moderate). Subgroup analysis showed similar results (Fig. 4).

## Discussion

Resectability of liver tumors is an evolving concept based on the possibility of radical resection of all tumor burdens with clear surgical margins. Therefore, MLR may potentially provide curative treatment for primary liver neoplasms, liver metastases, and tumors from other sites with a contiguous invasion of the liver.^17^^,^^33^, ^34^, ^35^

The short- and long-term outcomes of multivisceral resection have been studied for other gastrointestinal tumors, including colon, oesophageal, stomach, and pancreatic tumors.^36^, ^37^, ^38^ In a study from the present study's center, Dias et al.^36^ showed that multivisceral resection for gastric cancer is associated with higher perioperative complications (53.2% vs. 31.1%; p = 0.002) and shorter 5-year OS and DFS (55.4% vs. 71.5% [p < 0.001]; 51% vs. 77.8%; [p < 0.001], respectively) compared to a standard gastrectomy. Similarly, Petrucciani et al.,^38^ in a recent meta-analysis, showed that multivisceral pancreatic resection is associated with higher morbidity (56%‒69% vs. 37%‒50%) and mortality rates (10% vs. 4%) compared to a standard pancreatectomy.

MLR is still under debate because few studies have evaluated the impact of MLR on the outcomes of patients with malignant liver tumors. Although few studies have not found an impact of MLR on short- or long-term outcomes,^17^,^33^ other studies have reported a negative impact of MLR on perioperative results.^22^,^39^ Using a large database from the American College of Surgeons, Li et al.^39^ compared patients who underwent standard hepatectomy vs. en bloc hepatic and diaphragm resection due to several types of liver tumors, and they reported that the need for concomitant diaphragm resection is associated with a longer operative time, higher transfusion rate, longer length of hospital stay, higher overall morbidity, and higher frequency of major complications. Similarly, a recent matched case-control study (1:2) from the present group has reported that patients who undergo MLR have a longer operative time (430 vs. 360 min, p = 0.005), higher estimated blood loss (600 vs. 400 mL; p = 0.011), longer hospital stay (8 vs. 7 days; p = 0.003), and higher perioperative mortality (9.4% vs. 1.9%, p = 0.042). Importantly, the authors observed a higher density of deaths in the early time period after the resection, suggesting that the cumulative experience and improvements in perioperative care can decrease the mortality risk following MLR. Moreover, MLR does not negatively affect long-term outcomes.^22^ Therefore, an extended resection requires additional attention to postoperative complications and mortality, especially in the early time period after the resection; however, MLR may offer a valuable option of curative treatment for selected patients with locally advanced liver neoplasms.

The treatment of CRLM has largely evolved over the last decades, and it is currently based on the combination of modern systemic chemotherapy regimens and radical resection of liver lesions.^7^,^9^,^40^ The OS rates of patients with CRLM who underwent curative-intent hepatectomy have increased, reaching 40% to 60% at 5 years.^6^,^8^,^9^ In contrast, for patients with unresectable CRLM, the median OS is 18 to 36 months with palliative chemotherapy regimens.^41^,^42^ Negative surgical margins are associated with longer survival rates and a lower risk of local recurrence in patients with CRLM.^11^,^12^,^15^ Based on these premises, when a locally advanced CRLM involves an adjacent organ, liver resection combined with resection of the involved adjacent organ is required for oncologic curative resection.

However, the impact of MLR on patients with CLRM is still under debate due to several limitations of the available studies. The first is the rarity of these procedures even in high-volume referral centers. Hand et al.^17^ found 19 (3.6%) patients who underwent MLR out of 523 patients operated on for CRLM between 2005 and 2015. In the present center, the authors found 68 (11.2%) cases of MLR out of 609 patients operated on for CRLM over a 12-year period.^22^ Therefore, one of the major concerns about the studies addressing this issue is the small underpowered sample size. Another limitation is the lack of a standard definition of MLR. For this reason, the authors handled this potential bias using a clear definition of MLR derived from the definition used for multivisceral pancreatic surgery.^38^,^43^ Thus, MLR was defined as hepatectomy with en bloc resection of at least one adjacent organ or structure not usually removed in a standard procedure due to direct invasion by the liver tumor.^22^ Based on this definition, it is important to highlight that simultaneous resection of CRLM and the primary colorectal tumor were not considered an MLR.

Applying these criteria, the authors found only 9 comparative studies that assessed the outcomes of MLR in patients with locally advanced CRLM. Pooled analysis showed that MLR is associated with longer operative times, which is in line with other studies.^17^,^30^ Aoki et al.^30^ showed that patients who undergo MLR due to IVC or hepatic venous confluent invasion required almost double the time for resection compared to patients who undergo a standard hepatectomy (600 vs. 320 minutes; p < 0.001).

Despite technical difficulties, no differences in terms of estimated blood loss or transfusion rate were observed. Moreover, no increase in the length of hospital stay was found, except in the subgroup of patients with associated diaphragmatic resection. Other researchers who exclusively studied combined liver and diaphragmatic resections have reported conflicting results.^19^,^39^

Some studies have shown an increase in the perioperative complication rate in MLR; however, most of these studies included several different aetiologies in the same group.^22^,^34^ Conversely, the present meta-analysis demonstrated that MLR did not increase the overall morbidity in patients with CRLM, which agreed with Li et al.,^19^ who compared patients with CRLM who underwent hepatectomy and diaphragmatic resection vs. standard hepatectomies and did not find a significant difference in terms of perioperative morbidity (44.1% vs. 29.9%; p = 0.085). Similarly, in a matched case-control study (1:2), Hand et al.^17^ compared patients with CRLM who underwent MLR to those who underwent isolated hepatectomy and found no increase in perioperative complication rates (26.3% vs. 35%, p = 0.90). Importantly, the authors found an absolute increment of 7% in postoperative major complications. This is an interesting finding because most of the available studies did not directly assess this specific endpoint**.**

No significant difference in postoperative 30-day mortality was found. None of the included studies showed an increase in perioperative mortality, reinforcing the safety of MLR in patients with CRLM.

Regarding oncological outcomes, no difference in the frequency of compromised surgical margins was found between the group, supporting the use of MLR because it offers a similar rate of R0 resections for patients with locally advanced CRLM. In the previous study, the authors found similar rates of negative resection margins in patients with CRLM who underwent MLR compared to those with underwent an isolated hepatectomy (91% vs. 82.8%, p = 0.723).^22^

The OS rate was similar between the groups, indicating that MLR may offer a unique and valuable option for potentially curative treatment of locally advanced CRLM. Similarly, Shinke et al.^20^ reported similar OS for patients who underwent MLR or standard hepatectomy for CRLM in a nonmatched comparative study. Recently, a matched cohort analysis study has also reported no significant difference in the 1-, 3-, and 5-year OS rates following multivisceral resection or standard hepatectomy (75% vs. 82.1%, 56.6% vs. 53.4%, and 25.7% vs. 30.3%, respectively; p = 0.78).^17^

The present meta-analysis had several limitations. First, there is a lack of a clear definition of MLR. Thus, the present study was designed to minimize this bias using a clear definition of MLR, excluding cases of non-contiguous resection and hilar resection.^22^ Other limitations included the small number of available studies and the observational design. Despite these drawbacks, the present study is the first meta-analysis to evaluate the short- and long-term results of MLR in patients with CRLM, including a significant number of patients in comparison groups. Therefore, the present findings are the best available because RCTs are still lacking. However, the present findings should be confirmed by larger well-designed studies.

## Conclusion

In conclusion, MLR is a safe and feasible procedure but has a higher risk of major perioperative complications. MLR does not negatively affect long-term outcomes, indicating that an extended resection is a valuable option for potentially curative treatment for patients with locally advanced CRLM.

## Data availability statement

The datasets generated during and/or analyzed during the present study are available from the corresponding author upon reasonable request.

## Ethics approval statement

Not applicable.

## Patient consent statement

Not applicable.

## Permission to reproduce material from other sources

Not applicable.

## Authors’ contributions

Sérgio Silveira Júnior, data curation; Francisco Tustumi, formal analysis; Daniel de Paiva Magalhães, investigation; Vagner Birk Jeismann, methodology; Gilton Marques Fonseca, writing ‒ original draft; Jaime Arthur Pirola Kruger, writing ‒ review and editing; Fabricio Ferreira Coelho, conceptualization, supervision, and validation; Paulo Herman, conceptualization, validation, and data curation

## Funding statement

This research did not receive any specific grant from funding agencies in the public, commercial, or not-for-profit sectors.

## Declaration of Competing Interest

The authors declare that they have no known competing financial interests or personal relationships that could have appeared to influence the work reported in this paper.

## Appendix Supplementary materials

Image, application 1
