# Supplementary material for: The impact of multivisceral liver resection on short- and long-term outcomes of patients with colorectal liver metastasis: A systematic review and meta-analysis
Source: Clinics (Sao Paulo). 2022 Sep 17;77:100099. doi: 10.1016/j.clinsp.2022.100099 (PMC9489954; doi:10.1016/j.clinsp.2022.100099)
Supplement: Supplementary file 1 [file mmc1.docx]

**CLINICS-D-22-00057_Supplementary Material**

**Supplement File 1** Certainty of evidence assessment (GRADE).

| **Certainty assessment** | | | | | | |
| --- | --- | --- | --- | --- | --- | --- |
| **Participants (studies)** | **Risk of bias** | **Inconsistency** | **Indirectness** | **Imprecision** | **Publication bias** | **Overall certainty of evidence** |
|  |  |  |  |  |  |  |
| **30-days mortality** | | | | | | |
| 1786 (9 observational studies) | Serious^a^ | Not serious | Not serious | Not serious | None | ⨁⨁⨁◯ MODERATE |
| **Perioperative morbidity** | | | | | | |
| 1678 (8 observational studies) | Serious^a^ | Not serious | Not serious | Not serious | None | ⨁⨁⨁◯ MODERATE |
| **Perioperative major complications** | | | | | | |
| 484 (5 observational studies) | Serious^a^ | Not serious | Not serious | Not serious | None | ⨁⨁⨁◯ MODERATE |
| **Estimated blood loss** | | | | | | |
| 1678 (8 observational studies) | Serious^a^ | Very serious^b^ | Not serious | Serious^c^ | None | ⨁◯◯◯ VERY LOW |
| **Blood transfusion** | | | | | | |
| 748 (5 observational studies) | Serious^a^ | Very serious^b^ | Not serious | Not serious | None | ⨁◯◯◯ VERY LOW |
| **Compromised margins** | | | | | | |
| 1678 (8 observational studies) | Serious^a^ | Not serious | Not serious | Not serious | None | ⨁⨁⨁◯ MODERATE |
| **Operative time** | | | | | | |
| 1501 (8 observational studies) | Serious^a^ | Not serious | Not serious | Serious^d^ | None | ⨁⨁◯◯ LOW |
| **Length of hospital stay** | | | | | | |
| 1602 (7 observational studies) | Serious^a^ | Not serious | Not serious | Not serious | None | ⨁⨁⨁◯ MODERATE |
| **Overall survival** | | | | | | |
| 1786 (9 observational studies) | Serious^a^ | Not serious | Not serious | Not serious | None | ⨁⨁⨁◯ MODERATE |

^a^ Risk for selection bias.

^b^ I^2^ > 70%.

^c^ Confidence interval > 500 mL of blood.

^d^ Confidence interval > 60 min.

**Supplement File 2** Risk of bias assessment of included studies using ROBINS-I tool (Summary).

| **The risk of bias in non-randomized studies of interventions (ROBINS-I) assessment tool for cohort-type studies** | | | | | | | | |
| --- | --- | --- | --- | --- | --- | --- | --- | --- |
|  | **1. Bias due to confounding** | **2. Bias in selection of participants into the study** | **3. Bias in classification of interventions** | **4. Bias due to deviations from intended interventions** | **5. Bias due to missing data** | **6. Bias in measurement of outcomes** | **7. Bias in selection of the reported result** | **8. Overall bias** |
| **Hand et al.** | Low | Critical | Low | Low | Low | Low | Low | Moderate |
| **Shinke et al.** | Low | Critical | Low | Low | Low | Low | Low | Moderate |
| **Silveira Jr et al.** | Low | Critical | Low | Low | Low | Low | Low | Moderate |
| **Li et al.** | Low | Critical | Low | Low | Low | Low | Low | Moderate |
| **Lordan et al.** | Low | Critical | Low | Low | Moderate | Low | Low | Moderate |
| **Kazaryan et al.** | Low | Critical | Low | Low | Low | Low | Low | Moderate |
| **Lainas et al.** | Low | Critical | Low | Low | Low | Low | Low | Moderate |
| **Johnson et al.** | Low | Critical | Low | Low | Moderate | Low | Low | Moderate |
| **Aoki et al.** | Low | Critical | Low | Low | Low | Low | Low | Moderate |
